# The intrinsic value of choice: The propensity to under-delegate in the face of potential gains and losses

**DOI:** 10.1007/s11166-017-9259-x

**Published:** 2017-07-27

**Authors:** Sebastian Bobadilla-Suarez, Cass R. Sunstein, Tali Sharot

**Affiliations:** 1 Affective Brain Lab, Department of Experimental Psychology, University College London, WC1H 0AP, London, UK; 2 The Alan Turing Institute, 96 Euston Road, London, NW1 2DB UK; 3 Harvard Law School, Harvard University, Cambridge, MA USA

**Keywords:** Control premium, Delegation, Agency, Decision rights, Gains, Losses, C91, D03, D81

## Abstract

Human beings are often faced with a pervasive problem: whether to make their own decision or to delegate the decision task to someone else. Here, we test whether people are inclined to forgo monetary rewards in order to retain agency when faced with choices that could lead to losses and gains. In a simple choice task, we show that participants choose to pay in order to control their own payoff more than they should if they were to maximize monetary rewards and minimize monetary losses. This tendency cannot be explained by participants’ overconfidence in their own ability, as their perceived ability was elicited and accounted for. Nor can the results be explained by lack of information. Rather, the results seem to reflect an intrinsic value for choice, which emerges in the domain of both gains and of losses. Moreover, our data indicate that participants are aware that they are making suboptimal choices in the normative sense, but do so anyway, presumably for psychological gains.

## Introduction

In business, government, and daily life, people face a pervasive choice: whether to make a choice on their own or to delegate choice-making authority to someone else. With respect to investments, for example, one could rely on one’s own judgment, or one could rely instead on a trusted agent. Employees might choose health care plans on their own, or might ask employers to make the relevant choices. Patients and clients have the same dilemma in dealing with doctors and lawyers. Any principal can rely on or appoint an agent, who might have superior knowledge, might be immune from various biases, and might relieve the principal of the obligation to devote scarce time and limited cognitive resources to making difficult choices. On the other hand, an agent might have inferior knowledge, be ignorant of the principal’s real concerns, have her own biases, or be influenced by her own self-interest.

In theory, the decision whether to choose, or instead to delegate, should be a fully rational one, based on some form of cost-benefit analysis (Friedman 1953; Von Neumann and Morgenstern 2007). Choosers might begin by thinking in terms of expected value: would the payoff be higher with or without a delegation? They might also ask about the value of saving limited time and attention (McFadden 2001; Simon 1978). If the savings would be substantial, choosers might be willing to sacrifice something substantial in terms of expected value. It also matters whether choosing itself has benefits or costs, in the sense that choosers enjoy (or conversely dislike) the time that they devote to choosing. For some people, it may be interesting or fun to think about the best investments or the right health care plan. For other people, those choices are unpleasant and tiring, a kind of hedonic tax, and it is a great relief if someone else can make the choice for them.

Choosers might also consider whether the pleasure of a reward and the pain of a loss are amplified or reduced if they are personally responsible for the outcomes. Studies have shown that people value items they had selected themselves more than identical items that were selected for them (Brehm 1956; Egan et al. 2007, 2010; Lieberman et al. 2001; Sharot et al. 2009, 2010b). Neurologically, outcomes that were obtained by making an active choice are associated with greater activity in the striatum, a region that processes reward (Rao et al. 2008; Samejima et al. 2005; Sharot et al. 2009; Studer et al. 2012), and with heightened dopamine release (Syed et al. 2015), which is a neurotransmitter crucial for learning about the value of stimuli (Schultz et al. 1997). Thus, one could imagine a situation in which choosers would prefer (1) gaining $100 if that gain came from their own efforts to (2) gaining $110 if that gain came from someone else’s efforts, as the subjective value of self-attained $100 may be greater than that of $110 that was attained via an agent.

Consistent with this speculation, it has been found that people prefer options that permit further choice over those that do not (Bown et al. 2003; Catania 1981; Suzuki 1997). Similarly, people are willing to pay to control their own payoffs, rather than delegate, when faced with potential rewards (Owens et al. 2014). Would people also choose to pay to retain control in the face of potential loss? It is unknown whether choice has positive utility in situations where a person needs to decide between two aversive outcomes, such as when deciding between medical treatments or when deciding whether a stock should be sold or held to minimize loss. On the one hand, people may want to delegate choices that involve a potential loss as to avoid a feeling of regret for selecting the wrong option; in the same vein, regret avoidance may lead people to prefer to accept the status quo rather than make errors of commission (Samuelson and Zeckhauser 1988). On the other hand, a sense of control has been shown to reduce stress and anxiety in the face of unwanted outcomes (Alloy and Clements 1992; Shapiro et al. 1996; Thompson 1999). For that reason, making a choice may reduce the aversive utility of a loss (Sharot et al. 2010a) leading people to prefer agency over delegation.

Here, we test whether in the face of potential losses and gains, people will pay (or demand payment) to be choosers. On each trial, participants performed a simple choice between two shapes in order to maximize reward and minimize loss. On “gain trials,” a correct choice would result in a monetary gain and an incorrect choice in no gain. On “loss trials,” a correct choice would result in no loss and an incorrect choice in a monetary loss. After performing the task for an extended period of time on their own, participants were given an opportunity to delegate the decision making to an advisor. The expected value of using the advisor was disclosed on each trial and participants’ perception of their own expected value was also elicited. This allowed us to examine whether participants made “rational” delegation choices given their beliefs when faced with potential gains and with potential losses.

## Method

### Participants

Fifty-four participants (aged 18–61 years; mean age 25.0 years; 33 females) were recruited via a University College London website. Participants gave informed consent and were compensated for their time. Four participants were excluded from the analyses because they failed to complete all parts of the study. The study was approved by the University College London Research Ethics Committee. A summary of the instructions given to participants is provided in the Appendix.

### Stimuli

Stimuli included three hundred and sixty unique geometrical shapes varying in colour and orientation. Each shape appeared at most once throughout the study.

### Procedure

The study included two parts. Part I was a learning task and Part II was a delegation task. Each part consisted of one gain block (60 trials) and one loss block (60 trials). Order of gain and loss blocks was counterbalanced across participants.

### Part I: Learning task

**Fig. 1:**
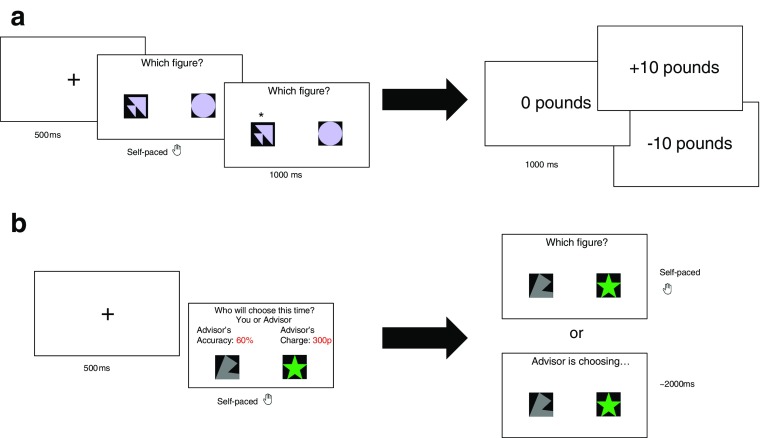
Task. **a** In the first part of the experiment—the Learning Task—participants were asked on each trial to choose between two shapes. If they successfully selected the shape associated with the better outcome they received £10 in the gain block and £0 in the loss block. If they were unsuccessful and selected the shape associated with the worse outcome they received £0 in the gain block and lost £10 in the loss block. **b** In the second part of the experiment—the Delegation Task—participants first chose whether they would like to retain agency and make the choice themselves or delegate the choice to an “artificial advisor” (a computer algorithm). They were presented with the advisor’s success rate and charge, and the pair of figures was shown at the time participants made the delegation choice. Outcomes were not revealed

The goal of the first part of the study was to familiarize the participants with a simple decision making task. On each trial, two shapes were presented and the participants’ task was to choose the shape that would deliver a better outcome. Participants were told that their task was to discover the rules, *if any*, that made some shapes “better” than others. No underlying rules governed winning shapes in our task. Rather, outcomes were random, such that each participant received the desirable outcome on 50% of the trials in each block. The pairs of shapes were drawn at random from the stimuli set without repetition and remained on screen until the participants made their decision. Once the choice was made, an asterisk (*) was shown above the chosen shape for one second and then a new screen with the outcome was presented for one second (**see** Fig. 1). In the gain block, the desired outcome was £10 and the undesired outcome was £0. In the loss block, the desired outcome was £0 and the undesired outcome was -£10. Each shape appeared only once throughout the experiment.

### Part II: Delegation task

The goal of Part II was to quantify participants’ willingness to delegate decisions to an advisor in the same task undertaken in Part I. On each trial, participants had to decide first whether to select between two novel shapes themselves or delegate the choice to an advisor. Participants were accurately informed that advisors were in fact computer algorithms (i.e. artificial agents), whose advice on each trial was determined prior to the start of the experiment. On each trial, there would be a different “artificial advisor.” On each trial, the pair of shapes was presented before participants had to decide whether to delegate the decision, so they had full information about the decision at hand.

On each trial, participants were presented with two pieces of information regarding the advisor: the advisor’s mean success rate in the task (from 0% to 100%, with mean 71.83%, SD = 23.9) and the advisor’s fee in British Pounds (from £0 to £10, with mean £3.44, SD = 3.55). Participants could decide either to delegate the decision between the shapes or to make the decision themselves. If participants decided to choose themselves, participants were given unlimited time to make their decision. If participants decided to delegate, a new screen appeared for approximately 2 seconds with the pair of novel shapes and text saying “Advisor is choosing….” (We emphasize that the participants were clearly instructed that the advisors were in fact algorithms and that the “decisions” of those algorithms were taken from a pool of past decisions.) In either case, participants would wait an additional one second before going on to the next trial. Outcomes (i.e. feedback) were not revealed, and thus participants were unable to learn about their own ability or to update their beliefs about the ability of the advisors. At the end of the study, ten trials would be chosen at random from Part II and the average outcome for those trials would be added to the baseline compensation of £7.

### Advisors

The participants paid the fee to the advisor only if they asked the advisor to choose for them and the advisor selected the correct shape. The expected value obtained by the participant by choosing to delegate ranged from £0 to £10 in the gain block with a mean of £5, and £0 to -£10 in the loss block with a mean of -£5. Because the participants’ objective accuracy rate was 50%, their expected value was £5 (=50% * £10) on a gain block and -£5 on a loss block. Thus, a value maximizer should choose to delegate whenever the advisor would return a mean expected value higher than £5 on a gain block but not otherwise (and be indifferent when the advisor’s expected value was exactly £5). On a loss block, a value maximizer should choose to delegate when the advisor would return a mean expected value that was higher than -£5 but not otherwise (and be indifferent when the advisor would return an expected value that was exactly -£5).

For example, on a gain block an advisor with 90% accuracy who charged £5 would return an expected value of ((accuracy*£10) – charge)) = ((90%*£10)-£5) = £4. On this trial, a value maximizer should not delegate. On half the trials, participants were offered advisors that would return an expected value greater than £5 (or greater than -£5 on loss trials) and the other half lower than £5 (or lower than -£5 on loss trials).

### Self-perceived accuracy (SPA)

At the end of the study, participants were asked to provide an estimate of how accurate they believed they were at choosing the correct shapes (from 1% to 99%). This estimate is referred to as self-perceived accuracy (SPA).

### Additional questions

To test whether the loss block was perceived negatively and the gain block positively (i.e. losses and gains are perceived as such), we asked participants at the end of the study which block (loss or gain) made them happier; which they would select to repeat; and how much they would pay to repeat each block.

### Gambling task

Participants completed an additional gambling task at the end of the experiment, which would enable us to estimate and account for risk attitudes when estimating the control premium in the gain and loss domain (task adapted from Charpentier et al. 2016). This is helpful, because the probability of the agent selecting the correct stimuli in relation to the participant doing so is different, and alters on each trial. Thus, risk preferences may influence delegation choices.

On each trial of the gambling task, participants selected between a sure option and a 50–50 gamble with varying values of gains and losses. On half the trials the sure option was £0, and the gamble option included gains ranging from £6 to £24 and losses ranging from £1 to £12. These trials allowed estimation of loss aversion. On half the trials, the sure option yielded a gain (range £1 to £12), and the gamble option included another gain (range £6 to £34) and £0. These trials allowed estimation of risk aversion (see Charpentier et al. 2016).

The task was divided into two blocks. The first block involved a staircase procedure that enabled estimation of the participant’s equivalence point between a gain-only gamble and £0. These gamble-related equivalence points were then used to construct trials in the second block for improved parameter estimation (see Charpentier et al. 2016). The staircase procedure involved updating the difference between the value of the sure option and the value of the gamble option every 2 trials, increasing amounts by £1 if the participant chose the gamble option, and decreasing amounts by £1 if the participant chose the sure option. The equivalence points were then used to generate 120 trials in the second block centred upon the equivalence point. At the end of the study, each participant’s reward for the whole experiment was revealed.

Three parameters were then estimated from the second block of trials in accordance with Prospect Theory equations (Kahneman and Tversky 1979): the loss aversion parameter λ, which is the ratio of sensitivity to losses to sensitivity of gains; the logit sensitivity μ, which is the consistency of participants’ choices across the task; and the risk aversion parameter ρ, which represents the diminishing sensitivity to changes in value as the absolute value increases. This is usually <1 for risk-averse individuals, and >1 for risk-seeking individuals. It is also the curvature of the utility function, assumed to be the same in gain and loss domains. These parameters were used to calculate the probability of accepting a gamble as per the following softmax function:

$$ P\left( gamble\; accepted\right)=\frac{1}{1+{e}^{-\mu \left(u(gamble)-u\left( sure\  option\right)\right)}} $$

where u(x) is the subjective utility of the respective options, estimated by:

$$ u(x)=\left\{\begin{array}{cc}\hfill \kern0.84em {x}^{\rho}\hfill & \hfill x>0\hfill \\ {}\hfill -\lambda {\left(-x\right)}^{\rho}\hfill & \hfill x<09\hfill \end{array}\right\} $$

Both the loss aversion parameter λ and the risk aversion parameter ρ would subsequently be used in our analysis of delegation indifference points (see below for more details on this analysis). These parameters would be included in our model as covariates.

### Analysis of delegation rates

We first calculated the percentage of trials in which participants chose to delegate. We then calculated the percentage of trials in which participants decided to delegate/retain agency out of all trials in which delegation was optimal (i.e. trials when the advisor’s expected value was above £5) and the percentage of trials in which they decided to delegate/retain agency when delegation was not optimal (i.e. trials when the advisor’s expected value was below £5). For loss trials this would be above and below -£5.

The same analysis was conducted considering the participants’ self-perceived accuracy (SPA). More specifically, we calculated the percentage of trials in which participants chose to delegate/retain agency when this was optimal given a participant’s SPA (i.e. the advisor’s expected value was above ((SPA*£10)-charge), and when it was suboptimal (i.e. below ((SPA*£10)-charge). For loss trials this would be above and below ((SPA*-£10)-charge).

### Analysis of indifference points

We calculated the indifference point of each participant as the expected value at which each participant would delegate with 50% probability. To this end, we ran a mixed effects model for each condition (Gains and Losses) separately with the advisor’s expected value as an independent variable, allowing for random effects and a random slope per participant. In other words, all participants had their own parameter estimates for the advisor’s expected value (modelled as a random slope) and for an intercept (also modelled as a random effect) drawn from a common Gaussian distribution for all participants (see Table 1). This type of model is appropriate for repeated measures because the regression coefficients are given their own probability model (Gelman and Hill 2007). They are also calculated more efficiently given that they are estimated hierarchically, both within and across participants (Bagiella et al. 2000; Clark and Linzer 2015; Gelman and Hill 2007).

**Table 1:** Mixed Effects Model of Delegations

Gains Model				
	*B*	*SE*	*z*	*p*
Fixed Effects				
(Intercept)	−8.44	1.26	−6.703	<.001
Expected Value	11.02	0.98	11.29	<.001
Trial Number	−0.009	0.005	−1.86	0.063
Rho	0.04	0.08	0.54	0.589
Lambda	−0.02	1.68	−0.01	0.991
Losses Model				
	*B*	*SE*	*z*	*p*
Fixed Effects				
(Intercept)	−6.42	1.05	−6.12	<.001
Expected Value	9.77	0.83	11.83	<.001
Trial Number	−0.02	0.005	−3.06	0.002
Rho	0.09	0.07	1.27	0.2
Lambda	−1.27	1.46	−0.87	0.38

Coefficients (*B*), standard errors (*SE*), *z* statistics and *p* values for the mixed effects model in the Gains condition (top) and the Losses condition (bottom). The results reported here are the fixed effects

We used the model predictions for all participants to estimate their indifference points. We controlled for trial number (to account for linear temporal effects such as fatigue or disengagement over time), loss aversion (λ), and risk aversion (ρ) in the model. Random effects were also included for trial number but not for the loss aversion and risk aversion parameters since these parameters were constant across observations within each participant. The latter two were estimated in a separate gambling task (see above). Median estimates of λ and ρ were 1.51 and 0.65, respectively.

### Analysis of control premiums

Because participants’ expected value was £5 in the gain block and -£5 in the loss block (their probability of choosing correctly was always 50%), a value maximizer’s indifference point would be £5 for gain and -£5 for loss if she had an accurate perception of her ability. Thus the “control premium”—the amount of money participants are willing to forgo to retain agency—would be equal to her indifference point minus £5 (or -£5 for loss). However, participants’ perception of their own ability is not completely accurate. Thus, if we take into account the participants’ perception of their own accuracy (SPA), the control premium is equal to their indifference point minus the SPA.

### Analysis of money forgone

To calculate the amount of money participants forgo in order to retain control, we calculated the average expected value for all of a participant’s choices, relative to the amount she could have received if she had selected to delegate optimally. This measure is different from the control premium described above, since it averages over all gains and losses for all actual choices throughout the experiment without estimating each participant’s indifference point.

## Results

### Delegation

Participants had a strong preference to retain agency. While a value maximizer would delegate 50% of the time, participants’ average delegation rate was significantly lower (mean delegation for Gains = 28.57%, one sample t-test compared to 50% *t*(49) = 8.84, *p* < 0.001; mean delegation for Losses = 29.27%, one sample t-test compared to 50% *t*(49) = 8.36, *p* < 0.001). Importantly, no differences were observed between Gains and Losses for this measure *t*(49) = 0.549, *p* = 0.586, or any other measure reported below. A Bayes factor analysis (Morey and Rouder 2011) was conducted to test further for null differences between the two conditions. This analysis outputs a ratio between the null hypothesis (i.e. conditions do not differ) and the alternative hypothesis (i.e. conditions differ). The analysis strongly supports the null hypothesis of no differences. It is 5.64 times more likely that the proportion of delegations was equal in the Gains and Losses conditions than that they were different.

### Delegation “errors”

**Fig. 2:**
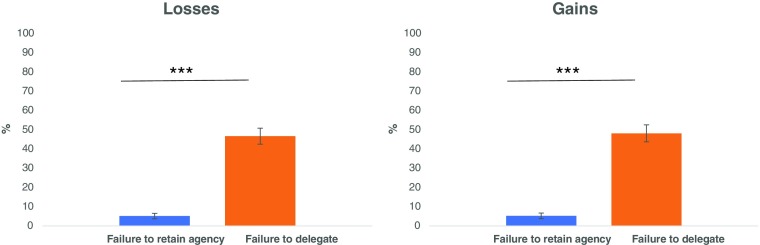
Delegation “Errors”. Participants were more likely to fail to delegate when delegation was optimal (*orange bars*) than fail to retain agency when retaining agency was optimal (*blue bars*). This was true for both blocks: (**a**) Gain block, (**b**) Loss block. ***p < 0.001. Error bars are standard errors of the mean

Participants were much more likely to retain agency when this was not the optimal decision (i.e., “*failure to delegate*”) than to delegate when this was not the optimal decision (i.e., “*failure to retain agency*”). Specifically, out of all trials where delegation was optimal, they chose to retain agency, *failing to delegate*, 47.33% in the gain block and 48.07% in the loss block (Fig. 2). In contrast, out of all trials where agency was the optimal decision they chose to delegate, *failing to retain agency*, only 5.2% in the gain block and 5.13% in the loss block (Fig. 2). As a result of these failures, participants earned £0.71 (SD = 0.495) less than they could have if they selected optimally in the gain block and lost £0.68 (SD = 0.467) more than they should have in the loss block.

**Fig. 3:**
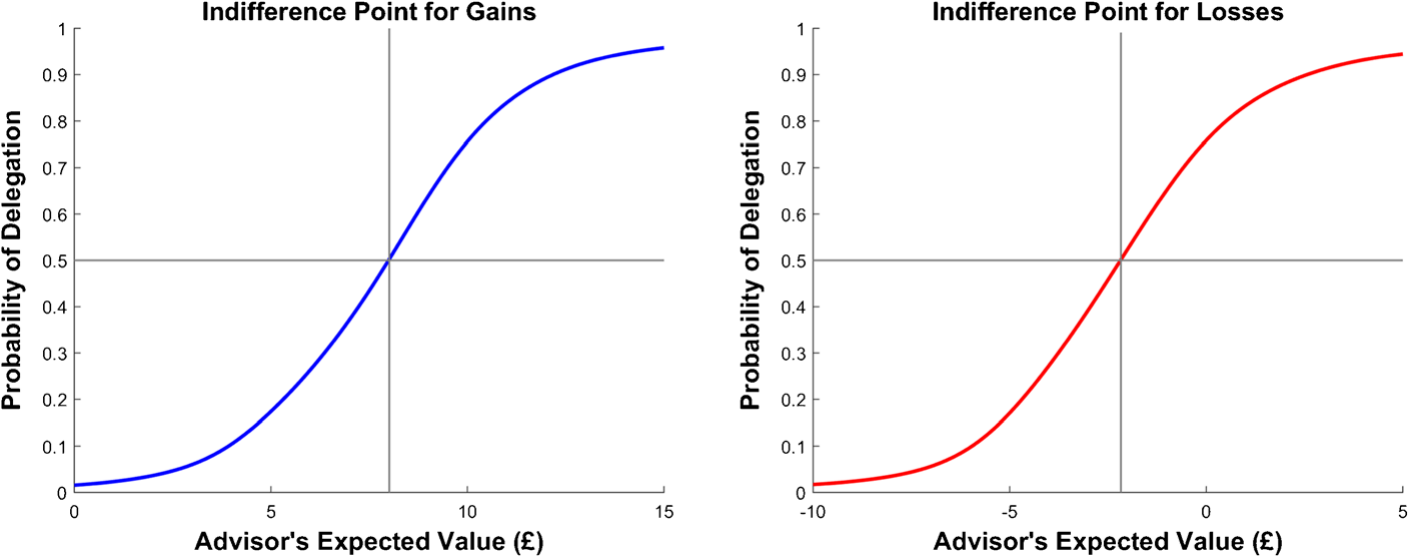
Indifference Points. The indifference point is the point when the probability of delegating is 50%. While a rational agent should be indifferent between retaining agency and delegating when an advisors’ expected value is £5 in the gain block and -£5 in the loss block (participant’s expected value is £5 in the gain block and -£5 in the loss block) the graphs clearly show that in practice the indifference point is greater than £5 in the gain block and -£5 in the loss block. This suggests that participants assign positive utility to choice. The grey lines show the intersection between 50% probability of delegation and the indifference curve. The curves shown here are the model predictions for the group average

Note, however, that while participants were not maximizing their rewards, they were not delegating at random. Rather, they were more likely to delegate on trials when delegation was optimal (51.93%) than on trials when delegation was not optimal (5.20%) *t*(49) = 11.40, *p* < 0.001) in both the gain block and in the loss block (53.4% and 5.13% respectively, *t*(49) = 11.77, *p* < 0.001), indicating that they were sensitive to the expected utility of delegating to the advisors (Fig. 3).

### Self-perceived accuracy (SPA)

Participants’ perceptions of their own ability to select the shape associated with the better outcome was relatively accurate (average estimate was 56.68%) but differed from the true performance rate of 50% (*t*(49) = 3.07, *p* = 0.003). Thus, participants’ preference for agency might be explained by overconfidence in their ability to choose accurately. To account for this, we redefined optimal delegation, considering each participant’s perception of their own accuracy (see methods).

Even when taking this into account, participants were still more likely to retain agency on trials when delegation was in fact optimal (*failure to delegate* on gain block = 44.07%, loss block = 42.27%) than to delegate when retaining agency was in fact optimal (*failure to retain agency* on gain block = 8.73%, loss block = 7.87%). The frequency of failing to delegate was significantly different from the frequency of failing to retain agency (Gain: *t*(49) = 5.91, *p* < 0.001; Loss: *t*(49) = 6.74, *p* < 0.001).

### Indifference point & control premium

When would participants be indifferent between making the decision themselves and letting the advisor make it for them? A rational agent should be indifferent between delegating the choice and retaining agency when the advisor’s expected value is £5 in the gain block and -£5 in the loss block. This is because the participant’s own expected value is £5 for gains and -£5 for losses. Thus, in those situations it should not matter who makes the decision.

We compare this benchmark to the indifference points that were observed given our participants’ responses (except for one participant for whom we could not estimate an indifference point given the low variance in that participant’s responses in the Gains condition). We observed that our participants’ average indifference point was £8.15 (SD = 2.92) in the gain block and -£0.822 (SD = 7.23) in the loss block, which is significantly different from £5: gains: *t*(48) = 7.55, *p* < 0.001, and -£5: loss: *t*(49) = 4.05, *p* < 0.001. In other words, participants were willing to forgo £3.15 (i.e. = £8.15 - £5) (SD = 2.92) in the gain block and lose an extra £4.18 (i.e. = − £0.822 - (−£5)) (SD = 7.23) in the loss block in order to retain agency.

We refer to this number as a control premium. Re-calculating the control premium, considering participants’ self-perceived accuracy (SPA) by subtracting the SPA of each participant from their indifference point, results in the following control premium estimates: £2.50 (SD = 3.18) for gains and £3.53 (SD = 6.81) for losses. These control premiums are significantly different from the previous estimates, for both gains and loss: *t*(48) = 2.94, *p* = 0.005. Even though controlling for SPA reduces the control premium estimate, the estimate is still much greater than zero, gains: *t*(48) = 5.50, *p* < 0.001, and losses: *t*(49) = 3.63, *p* = 0.001. Thus, the control premium seems to stem from an intrinsic value for control.

### Perceived delegation accuracy

In addition to asking participants to report self-perceived accuracy of choosing the correct shapes, we asked them to estimate how accurate they were at delegating. Participants’ estimates were surprisingly accurate and did not differ significantly from how well they were in fact delegating. The latter was calculated as the percentage of trials on which they made optimal decisions (i.e. delegating when they should be and retaining agency when they should be). Comparing perceived delegation performance and actual delegation performance showed no difference for gains: *t*(49) = 0.04, *p* = 0.97, or losses, *t*(49) = 0.30, *p* = 0.77. Neither did perceived accuracy of delegation and actual accuracy of delegation differ when optimal delegations were defined taking into account SPA (gains, *t*(49) = 0.12, *p* = 0.90, losses, *t*(49) = 0.69, *p* = 0.50).

The two scores correlated with each other; in other words, participants’ perceived accuracy of delegation correlated with actual accuracy of delegation defined based on SPA (loss: *r*(48) = 0.38, *p* = 0.007, marginally for gains: *r*(48) = 0.27, *p* = 0.054). SPA and perceived accuracy of delegation were also correlated (*r*(48) = 0.49, *p* < 0.001). Together, these results suggest that participants knew how well they were delegating and were aware that by retaining control they were losing money—yet chose to do so nevertheless.

### Loss and gain questions

To show that the participants made an explicit distinction between loss and gain blocks, we asked (i) which block made them happier—100% of participants responded that they were happier during the gain block than loss block; and (ii) which block they would prefer to do again—96% selected the gain block. In addition, (iii) 92% of participants would pay more money to repeat the gain block over the loss block.

## Discussion

Our results demonstrate that participants are willing to forgo rewards for the opportunity to make their own choices and hence to control their own payoffs. This preference was observed not only when faced with potential gains (in accord with Owens et al. 2014) but also when faced with potential losses. Moreover, our findings indicate that participants are aware of the (sub)optimality of their delegation choices, suggesting that they are also aware of the premium they are paying to maintain control.

The results could not be accounted for by participants’ overconfidence in their ability to maximize rewards and minimize losses, as their beliefs regarding their own ability were elicited and accounted for with self-perceived accuracy (SPA). (On average, participants were slightly overconfident in the learning task even though they were unlikely to hold prior beliefs about their ability from “real world” experience and were given plenty of experience with the task.) Participants were also given complete information about potential advisors, which would allow them to make rational decisions. Thus, under-delegation could not be attributed to a systematic misperception of either the participant’s expected utility or the advisor’s expected utility. Indeed, when questioned about their ability to delegate accurately, participants provided surprisingly accurate assessments (despite slight overconfidence in the learning task).

Our findings are in accord with a past study that identified a significant “control premium” in an experimental setting in which participants could bet that a partner, or instead they themselves, would answer quiz questions correctly (Owens et al. 2014). In light of participants’ elicited beliefs, participants should have bet on themselves 56.4% of the time—but in fact did so 64.9% of the time. It follows that participants were, on average, willing to give up 8% to 15% of their expected earnings to retain control. As in our study, the preference for control could not simply be explained by overconfidence or subjective beliefs; control appeared intrinsically desirable.

Fehr et al. (2012) also find, in a fundamentally different design, that people will sacrifice their material interest to maintain authority. They conduct an “authority game,” in which principals could choose to delegate decisions to an agent. Their central finding is that people will under-delegate, showing “a strong behavioral bias among principals to retain authority against their pecuniary interests and often to the disadvantage of both the principal and the agent.” Their major explanation is that people do not like to be overruled, and they know that if they delegate, their agents might disregard their information, or their wishes, in order to make their own selection. Using a different experimental design, Bartling et al. (2013) similarly find that decision rights have intrinsic and not merely instrumental value. There is also a relationship here between our findings and the phenomenon of “reactance,” which suggests that people rebel against choice-denying commands by defying them (Brehm and Brehm 2013).

Our results support the past findings for the existence of a “control premium” and extend them by demonstrating a positive control premium not only in the domain of potential gains but also in the domain of potential losses. Furthermore, our results suggest that participants are aware that they are choosing to pay a premium for control.

Why would people choose to under-delegate when they are aware of the material cost of under-delegation? This behavior reflects a non-monetary intrinsic value for control, which is expressed via choice. And while further work would be most valuable on this question, we speculate that the intrinsic value of choice may have emerged for three reasons.

First, outcomes that we select ourselves often suit our preferences and needs more than those that have been selected for us (Beattie et al. 1994; Kray 2000; Polman 2010; Stone and Allgaier 2008; Waldfogel 1993). This idea, fundamental to the liberal political tradition, helps to undergird the idea that so long as the interests of third parties are not at stake, people should be able to make choices for themselves, because they best know what will improve their welfare. On this view, the intrinsic value of choice operates as a kind of heuristic—one that usually or often works well, but that can also produce serious mistakes.

In support of that heuristic, environments in which we can and do exercise choice are usually (of course not always) more rewarding to us (Leotti et al. 2010; Patall et al. 2008; Rotter 1966). The frequent association between the exercise of choice and the existence and magnitude of reward may have led choice itself to be experienced as rewarding—something we seek and enjoy. So too, people might generally treat choice as intrinsically valuable because doing so usually leads to higher rewards. To be sure, findings about behavioral biases have raised many questions about the claim that choosers are always the best judges of what will promote their welfare, but perhaps the claim has sufficient truth to justify according intrinsic value to control and choice (Waldfogel 1993).

Second, a biological system that provides higher intrinsic reward for things we have obtained ourselves compared to things that were chosen for us may be adaptive, because it promotes learning. If we learn that an action results in a reward, we can repeat that action in the future to gain more of the same. If, by contrast, we do not execute an action to obtain reward (or avoid harm), we lose the opportunity to acquire a “blueprint” of how to gain rewards (or avoid harm) again in the future. Thus, the value of outcomes we have obtained ourselves emerges both from their utility *and* from the information they offer for future outcomes.

Third, aversion to ambiguity may contribute to the preference for control. It is possible that people perceive more ambiguity when they ask what will happen with delegation than when they retain control. Thus, risk ambiguity could help to generate the observed behavior.

We emphasize that the value of choice is likely to vary across persons, cultures, norms, and contexts. For choices that are especially fun or enjoyable to make, and when people want to be personally responsible for outcomes, the value of retaining choice will increase. When choosing is unpleasant, or when people do not want to be personally responsible, the value will decrease, and delegation itself might turn out to have value.

## Conclusion

In this controlled laboratory experiment, we show that people are willing to pay a control premium to make their own choices. Consistent with previous work, but with a somewhat different design, we find that people will pay a control premium in the domain of gains, and we also report a similar premium in the domain of losses. This finding runs counter to the idea that people prefer to delegate decisions involving unwanted outcomes in order to avoid regret (Loomes and Sugden 1982) and instead supports the notion that in important contexts, choice may be preferred regardless of expected valence of the outcome (Cockburn et al. 2014; Leotti and Delgado 2011, 2014; Sharot et al. 2009, 2010a). Moreover, the current study suggests that people are aware of the monetary premium they are paying to retain agency, but they do so anyway, presumably for psychological benefit. Thus, in the normative sense, choosers can be losers, and knowingly so.

## Appendix

### Summary of instructions given to participants

This experiment consists of 3 stages:

The first stage of the experiment is a training task. On each trial two shapes will be presented and your task will be to choose the shape that will deliver a better outcome. Your task will be to discover the rules, if any, that make some shapes better than others. There are two kinds of trials in this part of the experiment. In one kind of trial you will have the opportunity of winning 10 pounds or nothing. In the other type of trial, you will either lose 10 pounds or not lose any money at all. Instructions will be given onscreen, including which buttons you need to press during the experiment. I (the experimenter) will be outside should you have any questions during the task.

The second stage of the experiment is divided into two parts. On each trial, you will decide first whether to select between two shapes or to delegate the choice to an advisor. It is important to mention that these advisors are in fact artificial agents, whose advice was determined by a computer algorithm prior to the start of the experiment. On each trial, there will be a different advisor and the advice they offer is drawn from the responses that have already been computed previously. You will be presented with two pieces of information regarding the advisor: the advisor’s success rate in the task and their fee. You will only pay this fee if the advisor selects the correct shape. If you choose the advisor, then the corresponding advisor will make the choice for you. If you decide to make the choice yourself, then you will just choose between the shapes as you did in the first stage of the experiment. It is also important to mention that the choices you make in this second part of the experiment will have an impact on your final compensation. Ten trials will be sampled at random and (depending on the choices you made) we will average the winnings and losses and add that to your final compensation. Other instructions, including which buttons you need to press during the experiment, will be given onscreen. I (the experimenter) will be outside should you have any questions during the task.

The third stage of the experiment is a gambling task. In this task, you must decide whether you want to participate in a 50–50 gamble or choose the “sure” option. The sure option will normally be an amount of money that is lower than what could be gained with the gamble option. Similarly, as in the second task, ten trials will be sampled at random and we will average the winnings and losses to add that to your final compensation. Other instructions, including which buttons you need to press during the experiment, will be given onscreen. I (the experimenter) will be outside should you have any questions during the task.
